# Supplementary material for: Emergence of New SARS-CoV2 Omicron Variants after the Change of Surveillance and Control Strategy
Source: Microorganisms. 2022 Sep 30;10(10):1954. doi: 10.3390/microorganisms10101954 (PMC9610377; doi:10.3390/microorganisms10101954)
Supplement: Supplementary file 1 [file microorganisms-10-01954-s001.zip › Supplemetary Table S1.pdf]

Supplementary Table S1. Data on age, sex, pangolin lineage and origin were collected them, from swabs selected to be classified by WGS method.

gisaid_epi_isl	date	age	sex	pangolin_lineage	originating_lab
EPI_ISL_10070555	2022-01-17	54	Female	AY.119	HUCA
EPI_ISL_10070556	2022-01-28	90	Female	BA.2	H. JOVE
EPI_ISL_10070557	2022-02-03	88	Female	BA.1.1.1	HUCA
EPI_ISL_10070558	2022-02-04	40	Male	BA.2	HUCA
EPI_ISL_10070559	2022-02-05	45	Female	BA.1.1	HUCA
EPI_ISL_10070560	2022-02-07	26	Female	BA.2	HUCA
EPI_ISL_10070561	2022-02-08	54	Female	AY.119	HUCA
EPI_ISL_10070562	2022-02-08	56	Male	BA.1.1.1	HUCA
EPI_ISL_10070563	2022-02-09	27	Male	BA.1.1	IO FERNANDEZ VEGA
EPI_ISL_10070564	2022-02-09	87	Female	BA.1	H. MONTE NARANCO
EPI_ISL_10070565	2022-02-09	86	Female	BA.1.1.1	HUCA
EPI_ISL_10070566	2022-02-09	24	Male	BA.2	HUCA
EPI_ISL_10070567	2022-02-09	26	Female	BA.2	HUCA
EPI_ISL_10070568	2022-02-09	24	Female	BA.1.17	HUCA
EPI_ISL_10070569	2022-02-09	0	Female	BA.2.3	HUCA
EPI_ISL_10070570	2022-02-10	48	Female	BA.1.1.1	HUCA
EPI_ISL_10070571	2022-02-10	16	Female	BA.1	HUCA
EPI_ISL_10070572	2022-02-10	41	Male	BA.1.17	HUCA
EPI_ISL_10070573	2022-02-10	16	Female	BA.1.1	HUCA
EPI_ISL_10070574	2022-02-10	75	Male	BA.1.1	HUCA
EPI_ISL_10070575	2022-02-10	20	Female	BA.2.3	HUCA
EPI_ISL_10070576	2022-02-10	12	Female	BA.1.1	HUCA
EPI_ISL_10314325	2022-02-10	23	Female	BA.1.17	HUCA
EPI_ISL_10314326	2022-02-09	81	Male	BA.1	HUCA
EPI_ISL_10358440	2022-02-16	60	Female	BA.2.9	H. del Oriente de Asturias
EPI_ISL_10358441	2022-02-18	93	Male	BA.1.17	H. Jove
EPI_ISL_10358442	2022-02-10	48	Female	BA.2.3	HUCA
EPI_ISL_10358443	2022-02-11	45	Female	BA.2.3	HUCA
EPI_ISL_10358444	2022-02-12	36	Female	BA.2	HUCA
EPI_ISL_10358445	2022-02-12	42	Female	BA.1.17	HUCA
EPI_ISL_10358446	2022-02-12	73	Male	BA.1.17	HUCA
EPI_ISL_10358447	2022-02-14	48	Female	BA.2	HUCA
EPI_ISL_10358448	2022-02-15	26	Female	BA.2	HUCA
EPI_ISL_10358449	2022-02-19	83	Male	BA.2	HUCA
EPI_ISL_10358450	2022-02-20	51	Female	BA.1.1.1	HUCA
EPI_ISL_10358451	2022-02-20	73	Female	BA.1.1	HUCA
EPI_ISL_10358452	2022-02-20	58	Female	BA.1.1	HUCA
EPI_ISL_10551553	2022-02-10	35	Male	BA.2.3	HUCA
EPI_ISL_10551554	2022-02-11	22	Male	BA.2	HUCA
EPI_ISL_10551555	2022-02-11	53	Male	BA.2	HUCA
EPI_ISL_10551556	2022-02-16	41	Female	BA.2.3	HUCA
EPI_ISL_10551557	2022-02-16	14	Male	BA.2	HUCA
EPI_ISL_10551558	2022-02-18	52	Male	BA.1	HUCA
EPI_ISL_10551559	2022-02-18	24	Female	BA.2	HUCA
EPI_ISL_10551560	2022-02-19	9	Female	BA.1.1.14	HUCA
EPI_ISL_10551561	2022-02-19	69	Female	BA.1.1.1	HUCA
EPI_ISL_10551562	2022-02-20	18	Female	BA.1.1.1	HUCA
EPI_ISL_10615735	2022-02-17	80	Male	BA.1.1	H. JOVE
EPI_ISL_10615736	2022-02-19	17	Female	BA.2	HUCA
EPI_ISL_10647410	2022-02-18	13	Male	BA.1.1	HUCA
EPI_ISL_10647411	2022-02-18	39	Male	BA.2	HUCA
EPI_ISL_10647412	2022-02-19	52	Male	BA.1.1.1	HUCA
EPI_ISL_10647413	2022-02-19	75	Female	BA.2.3	HUCA
EPI_ISL_10647414	2022-02-21	39	Female	BA.1.1	HOSPITAL DE CABUEÑES
EPI_ISL_10647415	2022-02-21	90	Female	BA.1.1	HOSPITAL DE CABUEÑES
EPI_ISL_10647416	2022-02-20	64	Male	BA.2.9	H. DEL ORIENTE DE ASTURIAS
EPI_ISL_10647417	2022-02-21	41	Male	BA.2.3	HUCA
EPI_ISL_10647418	2022-02-23	27	Female	BA.2	HUCA

EPI_ISL_10647419	2022-02-23	19	Female	BA.2.12	HUCA
EPI_ISL_10647420	2022-02-23	36	Female	BA.2.9	HUCA
EPI_ISL_10647421	2022-02-23	38	Female	BA.2.3	HUCA
EPI_ISL_10688166	2022-02-21	1	Female	BA.2	HOSP. VALLE DEL NALON
EPI_ISL_10688167	2022-02-21	28	Male	BA.2	HUCA
EPI_ISL_10688168	2022-02-22	50	Male	BA.1	HUCA
EPI_ISL_10688169	2022-02-23	65	Male	BA.1.1	HUCA
EPI_ISL_10688170	2022-02-23	92	Male	BA.1.1.1	HUCA
EPI_ISL_10688171	2022-02-23	90	Female	BA.1.1.1	HUCA
EPI_ISL_10688172	2022-02-23	18	Female	BA.1.1.1	HUCA
EPI_ISL_10688173	2022-02-22	95	Female	BA.1.1.14	HUCA
EPI_ISL_10688174	2022-02-23	60	Male	BA.1.1.1	HUCA
EPI_ISL_10688175	2022-02-22	83	Female	BA.1.1	HUCA
EPI_ISL_10688176	2022-02-23	51	Female	BA.2	HUCA
EPI_ISL_10903011	2022-02-25	62	Female	BA.1.1.1	HUCA
EPI_ISL_10903012	2022-03-01	69	Male	BA.1.1.14	HUCA
EPI_ISL_10903013	2022-03-01	38	Female	BA.1.1.18	H. DEL ORIENTE DE ASTURIAS
EPI_ISL_10903014	2022-03-01	32	Female	BA.1.17	HUCA
EPI_ISL_10903015	2022-03-02	71	Female	BA.1.1	HUCA
EPI_ISL_10903016	2022-03-02	98	Female	BA.1.1	HUCA
EPI_ISL_10903017	2022-03-02	44	Male	BA.2.9	HUCA
EPI_ISL_10903018	2022-03-02	68	Male	BA.1.17	HUCA
EPI_ISL_10903019	2022-03-02	89	Male	BA.1.1.1	HUCA
EPI_ISL_10903020	2022-03-02	64	Female	BA.1.1.1	HUCA
EPI_ISL_10903021	2022-03-02	49	Female	BA.1.1	HUCA
EPI_ISL_10903022	2022-03-02	46	Female	BA.2	HOSPITAL DE CABUEÑES
EPI_ISL_10903023	2022-03-02	96	Male	BA.2.3	HUCA
EPI_ISL_10903024	2022-03-02	78	Male	BA.1.1.1	HUCA
EPI_ISL_10903025	2022-03-02	35	Male	BA.1.1.1	HUCA
EPI_ISL_10903026	2022-03-02	66	Female	BA.2	HUCA
EPI_ISL_10903027	2022-03-02	87	Female	BA.2.9	HUCA
EPI_ISL_10903028	2022-03-02	2	Female	BA.1.8	HUCA
EPI_ISL_10903029	2022-03-02	25	Female	BA.2	H. DEL ORIENTE DE ASTURIAS
EPI_ISL_10903030	2022-03-02	92	Female	BA.1.1.1	H. DEL ORIENTE DE ASTURIAS
EPI_ISL_10903031	2022-03-03	93	Male	BA.2	HUCA
EPI_ISL_11030680	2022-03-01	76	Female	BA.1.1	HUCA
EPI_ISL_11030681	2022-03-01	78	Male	BA.1	H. DEL ORIENTE DE ASTURIAS
EPI_ISL_11030682	2022-03-02	78	Male	BA.1.1	HUCA
EPI_ISL_11030683	2022-03-02	47	Female	BA.1	HOSPITAL DE CABUEÑES
EPI_ISL_11030684	2022-03-02	48	Female	BA.2	HUCA
EPI_ISL_11030685	2022-03-02	11	Male	BA.1.17	HUCA
EPI_ISL_11030686	2022-03-02	68	Male	BA.1	HUCA
EPI_ISL_11162585	2022-03-01	83	Male	BA.2	HUCA
EPI_ISL_11162586	2022-03-04	44	Male	BA.1.1.14	HUCA
EPI_ISL_11162587	2022-03-04	26	Male	BA.1.17	HUCA
EPI_ISL_11162588	2022-03-04	23	Female	BA.2	HUCA
EPI_ISL_11162589	2022-03-05	85	Female	BA.2	HUCA
EPI_ISL_11162590	2022-03-05	59	Female	BA.2	HUCA
EPI_ISL_11162591	2022-03-05	56	Male	BA.1.17	HUCA
EPI_ISL_11162592	2022-03-07	56	Female	BA.2.3	HUCA
EPI_ISL_11162593	2022-03-07	15	Male	BA.1.1.14	HUCA
EPI_ISL_11162594	2022-03-08	63	Male	BA.2.3	HUCA
EPI_ISL_11162595	2022-03-10	67	Male	BA.1.1	H. DEL ORIENTE DE ASTURIAS
EPI_ISL_11162596	2022-03-10	29	Female	BA.2.12	H. DEL ORIENTE DE ASTURIAS
EPI_ISL_11162597	2022-03-12	42	Male	BA.2.3	HUCA
EPI_ISL_11162598	2022-03-12	67	Female	BA.2.3	HUCA
EPI_ISL_11162599	2022-03-12	93	Male	BA.2.9	HUCA

EPI_ISL_11162600	2022-03-12	27	Female	BA.2	HUCA
EPI_ISL_11162601	2022-03-12	15	Female	BA.2.3	HUCA
EPI_ISL_11162602	2022-03-12	30	Female	BA.2	HUCA
EPI_ISL_11162603	2022-03-12	54	Female	BA.2.12	HUCA
EPI_ISL_11162604	2022-03-12	26	Male	BA.2	HUCA
EPI_ISL_11162605	2022-03-12	30	Male	BA.2	HUCA
EPI_ISL_11162606	2022-03-12	73	Male	BA.1	HUCA
EPI_ISL_11353508	2022-03-14	71	Male	BA.1.1.1	H. DEL ORIENTE DE ASTURIAS
EPI_ISL_11353509	2022-03-14	82	Female	BA.1.1	HUCA
EPI_ISL_11353510	2022-03-15	63	Male	BA.1.1.1	HUCA
EPI_ISL_11353511	2022-03-15	75	Female	BA.2	HUCA
EPI_ISL_11353512	2022-03-15	62	Male	BA.2	HUCA
EPI_ISL_11353513	2022-03-15	44	Female	BA.2	HUCA
EPI_ISL_11353514	2022-03-15	54	Male	BA.1.1	HUCA
EPI_ISL_11353515	2022-03-15	67	Male	BA.2.9	HUCA
EPI_ISL_11353516	2022-03-15	46	Female	BA.2.9	HUCA
EPI_ISL_11353517	2022-03-15	50	Female	BA.1.17	HUCA
EPI_ISL_11353518	2022-03-15	15	Female	BA.2	HUCA
EPI_ISL_11353519	2022-03-15	49	Male	BA.2	HUCA
EPI_ISL_11353520	2022-03-15	14	Female	BA.2	HUCA
EPI_ISL_11353521	2022-03-15	46	Male	BA.2	HUCA
EPI_ISL_11353522	2022-03-15	90	Female	BA.2.9	HUCA
EPI_ISL_11353523	2022-03-15	65	Female	BA.2	HUCA
EPI_ISL_11353524	2022-03-15	90	Male	BA.2.3	HUCA
EPI_ISL_11353525	2022-03-15	68	Female	BA.2	HUCA
EPI_ISL_11353526	2022-03-15	98	Female	BA.2.3	HUCA
EPI_ISL_11353527	2022-03-15	15	Female	BA.2	HUCA
EPI_ISL_11353528	2022-03-14	91	Female	BA.1.17	HUCA
EPI_ISL_11353529	2022-03-14	93	Female	BA.1.17	HUCA
EPI_ISL_11353530	2022-03-15	77	Male	BA.2.3	HUCA
EPI_ISL_11353531	2022-03-15	95	Female	BA.1.17	HUCA
EPI_ISL_11353532	2022-03-15	12	Male	BA.2	HUCA
EPI_ISL_11353533	2022-03-16	72	Male	BA.2.3	HUCA
EPI_ISL_11353534	2022-03-16	13	Female	BA.2	HUCA
EPI_ISL_11376264	2022-03-15	53	Female	BA.2	HUCA
EPI_ISL_11755001	2022-03-17	44	Female	BA.2	HUCA
EPI_ISL_11755002	2022-03-18	54	Female	BA.2	HUCA
EPI_ISL_11755003	2022-03-21	75	Male	BA.2	HUCA
EPI_ISL_11755004	2022-03-21	79	Male	BA.2	HUCA
EPI_ISL_11755005	2022-03-21	44	Male	BA.2.9	H. DEL ORIENTE DE ASTURIAS
EPI_ISL_11755006	2022-03-21	75	Female	BA.2	HUCA
EPI_ISL_11755007	2022-03-22	52	Female	BA.1	HOSPITAL DE CABUEÑES
EPI_ISL_11755008	2022-03-22	36	Female	BA.2	HUCA
EPI_ISL_11755009	2022-03-21	82	Female	BA.2.3	HUCA
EPI_ISL_11755010	2022-03-23	1	Male	BA.2	HOSP. VALLE DEL NALON
EPI_ISL_11755011	2022-03-24	50	Female	BA.2	HUCA
EPI_ISL_11755012	2022-03-25	90	Male	BA.1.1	HUCA
EPI_ISL_11755013	2022-03-25	87	Male	BA.2	HUCA
EPI_ISL_11755014	2022-03-26	87	Female	BA.2	HUCA
EPI_ISL_11755015	2022-03-26	28	Male	BA.2	HUCA
EPI_ISL_12107389	2022-03-22	60	Female	BA.2	HUCA
EPI_ISL_12107390	2022-03-22	42	Male	BA.2	HUCA
EPI_ISL_12107391	2022-03-21	84	Female	BA.2.3	HUCA
EPI_ISL_12107392	2022-03-22	39	Male	BA.2	HUCA
EPI_ISL_12107393	2022-03-28	76	Female	BA.1.17	HUCA
EPI_ISL_12107394	2022-03-29	100	Female	BA.2	HUCA
EPI_ISL_12107395	2022-03-29	62	Female	BA.2.3	HUCA
EPI_ISL_12107396	2022-03-29	74	Male	BA.2	HUCA
EPI_ISL_12107397	2022-03-29	86	Female	BA.2.3	HUCA
EPI_ISL_12107398	2022-03-28	88	Female	BA.1.17	HUCA
EPI_ISL_12107399	2022-03-28	94	Male	BA.1.17	HUCA
EPI_ISL_12107400	2022-03-28	86	Female	BA.1.17	HUCA
EPI_ISL_12107401	2022-03-30	64	Male	BA.2	HUCA
EPI_ISL_12107402	2022-03-31	82	Female	BA.2	HUCA
EPI_ISL_12107403	2022-03-31	77	Male	BA.1.17	HUCA
EPI_ISL_12107404	2022-04-01	44	Female	BA.2	HUCA

EPI_ISL_12107405	2022-04-02	41	Male	BA.2	HUCA
EPI_ISL_12107406	2022-04-02	60	Male	BA.2.3	HUCA
EPI_ISL_12107407	2022-04-02	70	Male	BA.1.1	HUCA
EPI_ISL_12107408	2022-04-02	95	Male	BA.2	HUCA
EPI_ISL_12107409	2022-04-02	21	Female	BA.2.3	HUCA
EPI_ISL_12107410	2022-04-02	48	Female	BA.2	HUCA
EPI_ISL_12107411	2022-04-02	49	Female	BA.2	HUCA
EPI_ISL_12107412	2022-04-03	32	Female	BA.2.9	HUCA
EPI_ISL_12222104	2022-03-22	74	Male	BA.2	HUCA
EPI_ISL_12222105	2022-04-01	52	Male	BA.2	HUCA
EPI_ISL_12222106	2022-04-01	95	Male	BA.2.3	HUCA
EPI_ISL_12222107	2022-04-04	50	Female	BA.2.9	HUCA
EPI_ISL_12222108	2022-04-05	85	Female	BA.2	HUCA
EPI_ISL_12222109	2022-04-06	83	Female	BA.2.12	H. JOVE
EPI_ISL_12222110	2022-04-07	51	Male	BA.1.1	HOSPITAL CARMEN Y S. OCHOA
EPI_ISL_12222111	2022-04-08	1	Female	BA.2.3	HUCA
EPI_ISL_12222112	2022-04-08	95	Female	BA.2	HUCA
EPI_ISL_12222113	2022-04-08	92	Male	BA.2.3	HUCA
EPI_ISL_12222114	2022-04-08	46	Female	BA.2	H. DEL ORIENTE DE ASTURIAS
EPI_ISL_12222115	2022-04-08	65	Female	BA.2.3	H. DEL ORIENTE DE ASTURIAS
EPI_ISL_12222116	2022-04-08	91	Female	BA.2	HUCA
EPI_ISL_12222117	2022-04-08	76	Male	BA.2.3	HUCA
EPI_ISL_12222118	2022-04-08	69	Male	BA.2	HUCA
EPI_ISL_12222119	2022-04-09	52	Female	BA.2	HUCA
EPI_ISL_12222120	2022-04-09	37	Male	BA.1.1	HUCA
EPI_ISL_12222121	2022-04-09	47	Male	BA.2	HUCA
EPI_ISL_12222122	2022-04-09	82	Female	BA.2.3	HUCA
EPI_ISL_12222123	2022-04-09	0	Female	BA.2	HUCA
EPI_ISL_12222124	2022-04-09	70	Female	BA.2	HUCA
EPI_ISL_12222125	2022-04-09	67	Male	BA.1.1.1	HUCA
EPI_ISL_12222126	2022-04-10	3	Female	BA.2.9	HUCA
EPI_ISL_12222127	2022-04-10	70	Male	BA.2	HUCA
EPI_ISL_12222128	2022-04-10	73	Male	BA.2	HUCA
EPI_ISL_12222129	2022-04-10	71	Female	BA.2.3	HUCA
EPI_ISL_12502798	2022-04-11	65	Male	BA.1.1	HUCA
EPI_ISL_12502799	2022-04-19	9	Male	BA.2.3	HUCA
EPI_ISL_12502800	2022-04-18	82	Female	BA.2.12	HUCA
EPI_ISL_12502801	2022-04-18	98	Female	BA.2.12	HUCA
EPI_ISL_12502802	2022-04-18	98	Female	BA.2.12	HUCA
EPI_ISL_12502803	2022-04-18	83	Female	BA.2.12	H. JOVE
EPI_ISL_12502804	2022-04-19	15	Male	BA.2	HOSPITAL CARMEN Y S. OCHOA
EPI_ISL_12502805	2022-04-19	49	Female	BA.2	HUCA
EPI_ISL_12502806	2022-04-19	42	Female	BA.2	HUCA
EPI_ISL_12502807	2022-04-19	90	Female	BA.2	HUCA
EPI_ISL_12502808	2022-04-19	72	Male	BA.2.3	H. DEL ORIENTE DE ASTURIAS
EPI_ISL_12502809	2022-04-19	86	Male	BA.2.9	H. DEL ORIENTE DE ASTURIAS
EPI_ISL_12502810	2022-04-19	31	Female	BA.2.9	HUCA
EPI_ISL_12502811	2022-04-19	75	Male	BA.2.9	HUCA
EPI_ISL_12502812	2022-04-19	71	Female	BA.2	HUCA
EPI_ISL_12502813	2022-04-20	59	Female	BA.2	HUCA
EPI_ISL_12502814	2022-04-20	26	Female	BA.2.9	HUCA
EPI_ISL_12502815	2022-04-19	0	Male	BA.2.12	HUCA
EPI_ISL_12502816	2022-04-20	46	Female	BA.2	HUCA
EPI_ISL_12502817	2022-04-20	13	Female	BA.2.3	HUCA
EPI_ISL_12502818	2022-04-20	0	Male	BA.2.3	HOSPITAL CARMEN Y S. OCHOA
EPI_ISL_12502819	2022-04-20	78	Male	BA.2	HUCA
EPI_ISL_12502820	2022-04-20	29	Female	BA.2	HUCA
EPI_ISL_12502821	2022-04-20	45	Female	BA.2	HUCA

EPI_ISL_12502822	2022-04-20	62	Male	BA.2	HUCA
EPI_ISL_12502823	2022-04-20	32	Male	BA.2	HUCA
EPI_ISL_12502824	2022-04-19	59	Female	BA.2.9	HUCA
EPI_ISL_12502825	2022-04-21	87	Female	BA.2	HUCA
EPI_ISL_12508163	2022-04-09	87	Male	BA.2	HOSPITAL CARMEN Y S. OCHOA
EPI_ISL_12511465	2022-04-24	57	Male	BA.2	HUCA
EPI_ISL_12511466	2022-04-24	75	Male	BA.2	HUCA
EPI_ISL_12511468	2022-04-25	52	Female	BA.2	HUCA
EPI_ISL_12511469	2022-04-25	35	Female	BA.2	HUCA
EPI_ISL_12511470	2022-04-25	28	Male	BA.2	HUCA
EPI_ISL_12511471	2022-04-25	7	Male	BA.2.3	HUCA
EPI_ISL_12511472	2022-04-25	64	Male	BA.1.1.1	HUCA
EPI_ISL_12511473	2022-04-26	1	Male	BA.2	HUCA
EPI_ISL_12511474	2022-04-26	71	Female	BA.2.9	HUCA
EPI_ISL_12511475	2022-04-26	51	Female	BA.2	HUCA
EPI_ISL_12511476	2022-04-26	24	Female	BA.2	HUCA
EPI_ISL_12511477	2022-04-26	53	Female	BA.2	HUCA
EPI_ISL_12511478	2022-04-26	85	Female	BA.2	HUCA
EPI_ISL_12511479	2022-04-26	29	Female	BA.2	HUCA
EPI_ISL_12511480	2022-04-26	64	Male	BA.2	HUCA
EPI_ISL_12511481	2022-04-26	43	Male	BA.2.23	HUCA
EPI_ISL_12511482	2022-04-26	62	Female	BA.2	HUCA
EPI_ISL_12511484	2022-04-26	102	Female	BA.2	H. MONTE NARANCO
EPI_ISL_12511485	2022-04-26	91	Male	BA.2.9	HUCA
EPI_ISL_12511486	2022-04-26	49	Female	BA.2.3	HUCA
EPI_ISL_12511487	2022-04-26	75	Male	BA.2	HUCA
EPI_ISL_12511488	2022-04-26	92	Male	BA.2	HUCA
EPI_ISL_12511489	2022-04-26	64	Female	BA.2.9	HUCA
EPI_ISL_12511490	2022-04-26	85	Female	BA.2	HUCA
EPI_ISL_12511491	2022-04-27	49	Male	BA.2	HUCA
EPI_ISL_12511492	2022-04-27	91	Female	BA.2.9	H. MONTE NARANCO
EPI_ISL_12511493	2022-04-26	84	Female	BA.2	HUCA
EPI_ISL_12589393	2022-04-25	62	Female	BA.5	HUCA
EPI_ISL_12589394	2022-04-26	29	Male	BA.5	HUCA
EPI_ISL_12589395	2022-04-27	50	Male	BA.2	Arriondas
EPI_ISL_12589396	2022-04-27	79	Male	BA.2.9	Arriondas
EPI_ISL_12589397	2022-04-27	52	Male	BA.2.9	HUCA
EPI_ISL_12589398	2022-04-27	64	Female	BA.5	HUCA
EPI_ISL_12589399	2022-04-27	50	Female	BA.2.3	HUCA
EPI_ISL_12589400	2022-04-28	71	Female	BA.5	HUCA
EPI_ISL_12589401	2022-04-28	46	Male	BA.1.1.1	HUCA
EPI_ISL_12589402	2022-04-28	30	Female	BA.5	HUCA
EPI_ISL_12589403	2022-04-28	56	Female	BA.2	HUCA
EPI_ISL_12589404	2022-04-29	35	Female	BA.5	HUCA
EPI_ISL_12589405	2022-04-29	73	Male	BA.5	HUCA
EPI_ISL_12589406	2022-04-29	64	Male	BA.5	HUCA
EPI_ISL_12589407	2022-04-29	63	Male	BA.2	HUCA
EPI_ISL_12589408	2022-04-29	42	Female	BA.5	HUCA
EPI_ISL_12589409	2022-04-30	85	Male	BA.2	HUCA
EPI_ISL_12589410	2022-04-30	61	Male	BA.2	HUCA
EPI_ISL_12589411	2022-04-30	73	Male	BA.2	HOSPITAL CARMEN Y S. OCHOA
EPI_ISL_12589412	2022-04-30	87	Male	BA.2	HUCA
EPI_ISL_12589413	2022-04-30	89	Female	BA.2	HUCA
EPI_ISL_12829084	2022-05-03	50	Female	BA.2.9	HUCA
EPI_ISL_12829085	2022-05-05	85	Female	BA.2	H. MONTE NARANCO
EPI_ISL_12829086	2022-05-07	80	Female	BA.2	HUCA
EPI_ISL_12829087	2022-05-07	91	Male	BA.2	HUCA
EPI_ISL_12829088	2022-05-08	60	Female	BA.2.12	HUCA
EPI_ISL_12829090	2022-05-08	90	Female	BA.2	HUCA
EPI_ISL_12829091	2022-05-10	89	Female	BA.2	HUCA
EPI_ISL_12829092	2022-05-11	51	Male	BA.2.18	HUCA
EPI_ISL_12829093	2022-05-11	unknown	Female	BA.2	HUCA
EPI_ISL_12829094	2022-05-11	46	Female	BA.2	HOSPITAL DE CABUEÑES
EPI_ISL_12829095	2022-05-11	34	Male	BA.2	HUCA

EPI_ISL_12829096	2022-05-10	70	Female	BA.2	HUCA
EPI_ISL_12829097	2022-05-10	76	Female	BA.2	HUCA
EPI_ISL_12829098	2022-05-10	74	Female	BA.2	HUCA
EPI_ISL_12829099	2022-05-10	65	Male	BA.2	HUCA
EPI_ISL_12829100	2022-05-10	74	Male	BA.2	HUCA
EPI_ISL_12829101	2022-05-10	51	Male	BA.2	HUCA
EPI_ISL_12829102	2022-05-10	75	Male	BA.2	HUCA
EPI_ISL_12829103	2022-05-10	69	Female	BA.2	HUCA
EPI_ISL_12829104	2022-05-10	64	Male	BA.2	HUCA
EPI_ISL_12829105	2022-05-10	91	Female	BA.2	HUCA
EPI_ISL_12829106	2022-05-10	85	Female	BA.2	HUCA
EPI_ISL_12829107	2022-05-10	79	Male	BA.2	HUCA
EPI_ISL_12829108	2022-05-10	77	Female	BA.2	HUCA
EPI_ISL_12829109	2022-05-10	73	Female	BA.2	HUCA
EPI_ISL_12829110	2022-05-10	88	Female	BA.2	HUCA
EPI_ISL_12829111	2022-05-10	76	Male	BA.2	HUCA
EPI_ISL_12829112	2022-05-10	60	Male	BA.2	HUCA
EPI_ISL_12829113	2022-05-10	84	Female	BA.2	HUCA
EPI_ISL_12829114	2022-05-10	64	Male	BA.2	HUCA
EPI_ISL_12829115	2022-05-10	83	Male	BA.2	HUCA
EPI_ISL_12829116	2022-05-11	73	Male	BA.5	HUCA
EPI_ISL_12829117	2022-05-12	70	Female	BA.2	HOSPITAL CARMEN Y S. OCHOA
EPI_ISL_12829118	2022-05-12	69	Female	BA.2	HUCA
EPI_ISL_12829119	2022-05-12	55	Female	BA.2	HOSPITAL ALVAREZ BUYLLA
EPI_ISL_12829120	2022-05-12	66	Female	BA.2	HUCA
EPI_ISL_12829121	2022-05-13	71	Female	BA.2.9	C.S. LLANES
EPI_ISL_12829122	2022-05-13	56	Female	BA.4	HUCA
EPI_ISL_12829123	2022-05-14	87	Male	BA.2.9	HUCA
EPI_ISL_12829124	2022-05-14	48	Female	BA.2	HUCA
EPI_ISL_12829125	2022-05-14	48	Female	BA.2.9	HUCA
EPI_ISL_12829126	2022-05-14	69	Male	BA.2.12	HUCA
EPI_ISL_12829127	2022-05-14	88	Female	BA.2.18	HUCA
EPI_ISL_12829128	2022-05-14	37	Female	BA.2	HUCA
EPI_ISL_12829129	2022-05-14	59	Female	BA.2	HUCA
EPI_ISL_12829130	2022-05-14	29	Female	BA.2	HUCA
EPI_ISL_12829131	2022-05-14	0	Female	BA.2	HUCA
EPI_ISL_12829132	2022-05-14	87	Female	BA.2	HUCA
EPI_ISL_12829133	2022-05-15	61	Male	BA.2.18	HUCA
EPI_ISL_12829134	2022-05-15	55	Female	BA.2	HUCA
EPI_ISL_12829135	2022-05-15	65	Female	BA.2	HUCA
EPI_ISL_12829136	2022-05-15	76	Female	BA.2	HUCA
EPI_ISL_12829137	2022-05-15	71	Female	BA.2	HUCA
EPI_ISL_12829138	2022-05-15	35	Male	BA.2	HOSPITAL CARMEN Y S. OCHOA
EPI_ISL_12829139	2022-05-15	49	Female	BA.2.9	HOSPITAL CARMEN Y S. OCHOA
EPI_ISL_8818639	2022-01-05	31	Female	AY.4.2	H. DEL ORIENTE DE ASTURIAS
EPI_ISL_8818655	2022-01-04	41	Female	AY.43	HUCA
EPI_ISL_8818656	2022-01-04	63	Male	AY.4	HUCA
EPI_ISL_8818657	2022-01-05	31	Male	BA.1.17	HUCA
EPI_ISL_8818658	2022-01-05	33	Female	BA.1.17	HUCA
EPI_ISL_8818659	2022-01-08	88	Female	BA.1.17	HUCA
EPI_ISL_8818660	2022-01-08	81	Male	BA.1	HUCA
EPI_ISL_8818661	2022-01-08	47	Male	BA.1	HUCA
EPI_ISL_8818662	2022-01-08	10	Female	BA.1	HUCA
EPI_ISL_8818663	2022-01-08	41	Female	BA.1.17	HUCA
EPI_ISL_8818664	2022-01-08	26	Male	BA.1.17	HUCA
EPI_ISL_8818665	2022-01-08	57	Female	BA.1.1.1	HUCA
EPI_ISL_8818666	2022-01-08	46	Male	AY.43	HUCA
EPI_ISL_8818668	2022-01-07	2	Female	BA.1.17	HUCA
EPI_ISL_8818669	2022-01-07	3	Female	BA.1.17	HUCA
EPI_ISL_9093964	2022-01-11	75	Female	BA.1.17	HUCA
EPI_ISL_9093965	2022-01-12	11	Female	BA.1.17	HUCA
EPI_ISL_9093966	2022-01-12	43	Female	BA.1.1.1	HUCA

EPI_ISL_9093967	2022-01-12	38	Female	BA.1.17	HUCA
EPI_ISL_9093968	2022-01-12	34	Male	BA.1.17	HUCA
EPI_ISL_9093969	2022-01-12	15	Male	BA.1.1.1	HUCA
EPI_ISL_9093970	2022-01-12	34	Female	BA.1.1.1	HUCA
EPI_ISL_9093971	2022-01-12	48	Female	BA.1	HUCA
EPI_ISL_9093972	2022-01-13	91	Male	BA.1.1.1	HUCA
EPI_ISL_9093973	2022-01-13	5	Female	BA.1	HUCA
EPI_ISL_9093974	2022-01-13	24	Female	BA.1.17	Z.B.SALAS
EPI_ISL_9093975	2022-01-13	71	Female	BA.1.1.1	Z.B.SALAS
EPI_ISL_9093976	2022-01-13	33	Male	BA.1.1.1	Z.B.SALAS
EPI_ISL_9093977	2022-01-13	47	Male	BA.1.1.1	Z.B.SALAS
EPI_ISL_9093978	2022-01-13	56	Female	BA.1.17	HUCA
EPI_ISL_9093979	2022-01-13	34	Male	BA.1.17	HUCA
EPI_ISL_9093980	2022-01-13	74	Female	BA.1	HUCA
EPI_ISL_9093981	2022-01-13	40	Female	BA.1.1	HUCA
EPI_ISL_9093982	2022-01-13	31	Male	BA.1.17	HUCA
EPI_ISL_9093983	2022-01-13	36	Female	AY.124	HUCA
EPI_ISL_9093984	2022-01-13	8	Female	BA.1.17	HUCA
EPI_ISL_9093985	2022-01-13	72	Male	AY.121	HUCA
EPI_ISL_9093986	2022-01-13	15	Male	BA.1	HUCA
EPI_ISL_9093987	2022-01-13	3	Male	BA.1.1.1	HUCA
EPI_ISL_9093988	2022-01-13	4	Male	BA.1.17	HUCA
EPI_ISL_9093989	2022-01-13	10	Male	BA.1.1.1	HUCA
EPI_ISL_9093990	2022-01-13	8	Female	AY.43	HUCA
EPI_ISL_9251073	2022-01-19	44	Female	BA.2	HUCA
EPI_ISL_9396157	2022-01-08	64	Male	AY.43	HUCA
EPI_ISL_9396158	2022-01-08	2	Female	BA.1.1	HUCA
EPI_ISL_9396159	2022-01-08	1	Male	BA.1.1.1	HUCA
EPI_ISL_9396160	2022-01-10	71	Male	BA.1	H.Jove
EPI_ISL_9396161	2022-01-10	71	Female	AY.119	H.Jove
EPI_ISL_9396162	2022-01-12	48	Male	BA.1.17	HUCA
EPI_ISL_9396163	2022-01-12	46	Female	BA.1.17	HUCA
EPI_ISL_9396164	2022-01-12	36	Female	BA.1	HUCA
EPI_ISL_9396165	2022-01-19	25	Female	AY.43	HUCA
EPI_ISL_9396166	2022-01-19	37	Male	AY.119	HUCA
EPI_ISL_9396167	2022-01-20	40	Male	AY.43	HUCA
EPI_ISL_9396168	2022-01-20	31	Male	AY.43	HUCA
EPI_ISL_9396169	2022-01-20	72	Female	AY.119	HUCA
EPI_ISL_9396170	2022-01-20	50	Female	AY.43	HUCA
EPI_ISL_9510790	2022-01-10	55	Male	BA.1	H.Jove
EPI_ISL_9510791	2022-01-19	33	Female	BA.1.17	HUCA
EPI_ISL_9796229	2022-01-20	44	Female	BA.1.1.1	HUCA
EPI_ISL_9796230	2022-01-21	63	Female	BA.1.18	HUCA
EPI_ISL_9796231	2022-01-23	84	Female	BA.1.17	HUCA
EPI_ISL_9796232	2022-01-23	9	Male	BA.1	HUCA
EPI_ISL_9796233	2022-01-23	35	Male	BA.1.17	HUCA
EPI_ISL_9796234	2022-01-23	42	Male	BA.1.1.1	HUCA
EPI_ISL_9796235	2022-01-24	37	Female	AY.4.8	H. DEL ORIENTE DE ASTURIAS
EPI_ISL_9796236	2022-01-25	32	Male	BA.1.17.2	HUCA
EPI_ISL_9796238	2022-01-25	77	Male	BA.1.17	HUCA
EPI_ISL_9796239	2022-01-25	92	Female	BA.1	H. DEL ORIENTE DE ASTURIAS
EPI_ISL_9796240	2022-01-30	91	Male	BA.1.1.1	HUCA
EPI_ISL_9796241	2022-01-30	45	Female	BA.1	HUCA
EPI_ISL_9796242	2022-01-30	25	Female	BA.1.17.2	HUCA
EPI_ISL_9796243	2022-01-30	13	Male	BA.1.1.1	HUCA
EPI_ISL_9796244	2022-01-30	35	Female	BA.1.1.1	HUCA
EPI_ISL_9863944	2022-01-26	33	Male	BA.2	HUCA
EPI_ISL_9863945	2022-01-26	59	Female	BA.2	HUCA
EPI_ISL_9978940	2022-01-29	64	Female	AY.119	HOSPITAL SAN AGUSTIN
EPI_ISL_9978941	2022-01-15	38	Male	BA.2	HUCA
EPI_ISL_9978942	2022-01-27	75	Male	BA.2	HUCA
EPI_ISL_9978943	2022-02-02	31	Female	BA.2	HUCA
EPI_ISL_9978944	2022-02-02	23	Male	BA.2.3	HUCA
EPI_ISL_9978945	2022-02-04	14	Male	BA.2	HUCA
EPI_ISL_9978946	2022-02-02	63	Female	BA.1.17	HUCA
EPI_ISL_9978947	2022-01-25	29	Female	BA.1.1.1	HUCA
EPI_ISL_9978948	2022-01-26	34	Female	BA.1.17	HUCA

EPI_ISL_9978949	2022-01-26	48	Female	BA.1.17	HUCA
EPI_ISL_9978950	2022-01-26	74	Female	BA.1.1.1	HUCA
EPI_ISL_9978951	2022-01-28	9	Male	BA.1.1	HUCA
EPI_ISL_9978952	2022-01-28	80	Male	BA.1.1	HUCA
EPI_ISL_9978953	2022-01-31	69	Female	BA.1.17	HUCA
EPI_ISL_9978954	2022-01-31	40	Female	BA.1.17	HUCA
EPI_ISL_9978955	2022-02-01	40	Male	BA.1.18	HUCA
EPI_ISL_9978956	2022-02-01	76	Male	BA.1.1	HUCA
EPI_ISL_9978957	2022-02-02	3	Male	BA.1	HUCA
EPI_ISL_9978958	2022-02-02	72	Male	BA.1	H. DEL ORIENTE DE ASTURIAS
EPI_ISL_9978959	2022-02-03	93	Female	BA.1.1.1	HUCA
EPI_ISL_9978960	2022-01-31	92	Female	BA.1.1	HUCA
EPI_ISL_9978961	2022-02-02	6	Male	BA.1.1	HUCA
